# Assessment of Daily Life Physical Activities in Pulmonary Arterial Hypertension

**DOI:** 10.1371/journal.pone.0027993

**Published:** 2011-11-16

**Authors:** Vincent Mainguy, Steeve Provencher, François Maltais, Simon Malenfant, Didier Saey

**Affiliations:** Centre de Recherche de l'Institut Universitaire de Cardiologie et de Pneumologie de Québec, Université Laval, Québec, Canada; University of Giessen Lung Center, Germany

## Abstract

**Background:**

In pulmonary arterial hypertension (PAH), the six-minute walk test (6MWT) is believed to be representative of patient's daily life physical activities (DL_PA_). Whether DL_PA_ are decreased in PAH and whether the 6MWT is representative of patient's DL_PA_ remain unknown.

**Methods:**

15 patients with idiopathic PAH (IPAH) and 10 patients with PAH associated with limited systemic sclerosis (PAH-SSc) were matched with 15 healthy control subjects and 10 patients with limited systemic sclerosis without PAH. Each subject completed a 6MWT. The mean number of daily steps and the mean energy expenditure and duration of physical activities >3 METs were assessed with a physical activity monitor for seven consecutive days and used as markers of DL_PA_.

**Results:**

The mean number of daily steps and the mean daily energy expenditure and duration of physical activities >3 METs were all reduced in PAH patients compared to their controls (all p<0.05). The mean number of daily steps correlated with the 6MWT distance for both IPAH and PAH-SSc patients (r = 0.76, p<0.01 and r = 0.85, p<0.01), respectively.

**Conclusion:**

DL_PA_ are decreased in PAH and correlate with the 6MWT distance. Functional exercise capacity may thus be a useful surrogate of DL_PA_ in PAH.

## Introduction

Pulmonary arterial hypertension (PAH) is characterized by a progressive increase in pulmonary vascular resistance ultimately leading to right heart failure and death. Despite considerable improvements in long-term prognosis with the modern advances in PAH-specific therapies [1], most patients display persistent and significant dyspnea, exercise intolerance and poor health-related quality of life. Because of its discriminative properties in idiopathic PAH [2]–[6], exercise capacity has been used as the primary endpoint in most of the recent placebo-controlled randomized trials in PAH [1]. Exercise capacity is also considered as a benchmark for disease severity, prognosis and response to therapy in PAH, and its routine assessment is currently recommended at baseline and follow-up [3]–[7].

In PAH, exercise tolerance is most commonly assessed by the six-minute walk test (6MWT), with the assumption that the distance walked during this test is representative of patient's daily life activities [8]. However, the relationship between the distance walked during the 6MWT and the level of daily life physical activities (DL_PA_) has not been documented in PAH. Moreover, decreased level of DL_PA_ has been subjectively reported by PAH patients [9]. However, whether PAH patients are objectively less physically active to a level that may theoretically contribute to deconditioning remains unknown.

The aims of this study were to test the hypothesis that the level of DL_PA_ of PAH patients is markedly lower compared to sedentary healthy subjects, and that the 6MWT could be a useful surrogate of DL_PA_ among PAH patients.

## Methods

### Ethics statement

The institutional ethics committee (Comité d'éthique de la recherche de l'Institut de cardiologie et de pneumologie de Québec, protocol number: CÉR 20142) approved the research protocol and all subjects gave written consent prior to study enrolment.

### Subjects

Consecutive patients with idiopathic pulmonary arterial hypertension (IPAH, n = 15) and with pulmonary arterial hypertension associated with limited systemic sclerosis (PAH-SSc, n = 10) were recruited at the Institut Universitaire de cardiologie et de pneumologie de Québec. The definition of limited systemic sclerosis was based on Leroy criteria [10]. The PAH diagnosis was made according to recent guidelines [11]. All patients displayed significant PAH, defined as a mean pulmonary artery pressure >25 mmHg at rest with a pulmonary capillary wedge pressure <15 mmHg [12]. Recent right heart catheterization (<12 months) was used to described hemodynamic severity. Only patients with no change in their PAH therapy, in stable clinical condition over the last 4 months and belonging to functional classes II or III defined by the New York Heart Association (NYHA) functional classification were eligible. Exclusion criteria were as follow: (1) unstable PAH defined as recent syncope or NYHA functional class IV; (2) left ventricular ejection fraction <40% of predicted; (3) significant restrictive (more than minimal lung fibrosis on CT scan or total lung capacity <70% of predicted) or obstructive (FEV_1_/FVC <70%) lung disease. IPAH patients were individually matched for age, gender and body mass index (BMI) with healthy control subjects (CTRL, n = 15) recruited by advertisement in the community. Similarly, PAH-SSc patients were matched with patients with limited systemic sclerosis (SSc, n = 10) after excluding PAH (normal physical examination and pulmonary function tests, and systolic pulmonary arterial pressure by Doppler-echocardiography of 30 (6) mmHg). Neither PAH patients, SSc patients or healthy controls were enrolled in a structured physical activity program before or during the study. All the subjects were told to maintain their usual activities during the study. As the time of the year may greatly influence weekly energy expenditure, particularly in Northern climate countries, PAH patients and matched controls were concomitantly recruited.

### Study design

After reviewing the medical history and obtaining anthropometric measurements, a 6MWT was performed according to recent recommendations [8]. Individual results were compared to predicted values [13].

Subjects were then equipped with a physical activity monitor (SenseWear® Pro armband; BodyMedia Inc., Pittsburgh, PA, USA). This device, measuring 8.8×5.6×2.1 cm and weighing 82 g, was positioned on patients' right upper arm (*triceps brachii*) skin at midpoint between the acromion and the olecranon. The monitor contains a biaxial accelerometer (longitudinal and transverse) and multiple sensors (galvanic skin response, heat flux, skin temperature and near-body ambient temperature). While activity questionnaires often yielded to inaccurate physical activities assessments [14]–[15], this multiaxial device provides objective, accurate, individualized and detailed description of activity patterns such as time and intensity of physical activities and has been validated in diverse populations including patients with chronic diseases [14], [16]–[19]. The data provided by the sensors are stored minute by minute and the energy expenditure determination is based on specific algorithms. This device provides the energy expenditure (kilocalories) and the time spent (minutes) of physical activities above a pre-determined intensity level (*e.g.* metabolic equivalents [METs]) as well as the number of steps.

Each subject was instructed to wear the physical activity monitor for the entire day (except sleep time and while showering or bathing) for seven consecutive days. To ensure that the measured DL_PA_ characteristics were representative of patient's usual functional status, patients were asked to avoid unfamiliar activities and fill a physical activity journal log. DL_PA_ were defined a priori by: 1) the mean number of daily steps, 2) the mean daily energy expended during physical activities inducing a metabolic demand >3 METs [20], and 3) the mean daily duration spent in physical activities inducing a metabolic demand >3 METs. The American College of Sports Medicine suggests the definition of the metabolic demand representative of moderate activity to be age-dependent in healthy subjects in order to take into account the impact of normal aging on daily exercise level [21]. However, this definition may not be suitable for chronically diseased subjects. For this reason, a metabolic demand >3 METs was defined a *priori* as a marker of moderate activity in our patients, as previously done in similarly impaired patients with chronic respiratory diseases [18], [22]. DL_PA_ were analyzed using the SenseWear® Pro software version 6.1.0.1523 (BodyMedia Inc., Pittsburgh, PA, USA).

### Statistical analysis

Quantitative variables were expressed as mean (SD) and qualitative variables as proportion. Chi-square test or Fischer's exact were used to compare qualitative variables and one-way ANOVA was used to compare mean values for quantitative variables. *Posteriori* comparisons among the four groups were performed using the Tukey's technique. The normality and variance assumptions were verified with the Shapiro-Wilk and with Brown and Forsythe's variation of Levene's tests respectively. Pearson correlation coefficients corrected for multiple testing (Bonferroni) were used to evaluate relationships between the distance walked during the 6MWT and the three markers of DL_PA_ as well as the right atrial pressure and the cardiac index. The results were considered significant with p-values ≤0.05 and ≤0.01 when corrected for multiple testing. DL_PA_ characteristics were normalized for the time the physical activity monitor was worn. The data were analyzed using the statistical package program SAS 9.2 (Service Pack 4, SAS Institute Inc., Cary, NC).

## Results

### Subjects' characteristics

The characteristics of the study population are shown in Table 1. PAH patients were well matched with their respective controls for age, gender and BMI. They were treated with bosentan (n = 13), epoprostenol (n = 6), sildenafil (n = 2), sitaxsentan (n = 2), ambrisentan (n = 1) and calcium channel blockers (n = 1). Finally, 16 and 9 PAH patients were classified as NYHA functional class II and III, respectively.

**Table 1:** Characteristics of the study population.

	CTRL	IPAH	SSc	PAH-SSc
	n = 15	n = 15	n = 10	n = 10
**Demographics**				
Age (yr)	46 (16)	47 (15)	58 (9)	58 (10)
Sex (F/M)	(10/5)	(10/5)	(9/1)	(9/1)
BMI (kg/m^2^)	26 (5)	27 (8)	25 (5)	26 (5)
**Pulmonary hemodynamic**				
RAP (mm Hg)	NA	7 (2)	NA	5 (3)
mPAP (mm Hg)	NA	46 (13)	NA	41 (13)
PCWP (mm Hg)		10 (3)		9 (3)
CI (L/min•m^−2^)	NA	2.9 (0.8)	NA	3.1 (0.8)
PVRi (WU•m^2^)	NA	13.8 (6.4)	NA	10.5 (5.9)
**NYHA Functional class**				
(II/III)	NA	(11/4)	NA	(5/5)
**Exercise tolerance (6MWT)**				
Distance (m)	670 (64)	401 (89)*	502 (55)	349 (129)†
Distance (% pred)	120 (20)	75 (14) *	100 (10)	72 (21) †

Values are mean (SD).

*p<0.05 IPAH vs. CTRL.

† p<0.05 PAH-SSc vs. SSc.

*Table legend:* yr, years; F, female; M, male; BMI, body mass index; kg, kilogram; RAP, right atrial pressure; mPAP, mean pulmonary artery pressure; PCWP, pulmonary capillary wedge pressure; CI, cardiac index; PVRi, pulmonary vascular resistance index; WU, Wood units; NYHA, New York Heart Association; 6MWT, six-minute walk test; m, meters; % pred, percentage of predicted value; CTRL, healthy control subjects; IPAH, patients with idiopathic pulmonary arterial hypertension; SSc, subjects with limited systemic sclerosis without PAH; PAH-SSc, patients with pulmonary arterial hypertension associated with limited systemic sclerosis.

### DL_PA_ characteristics and functional status

**Figure 1:**
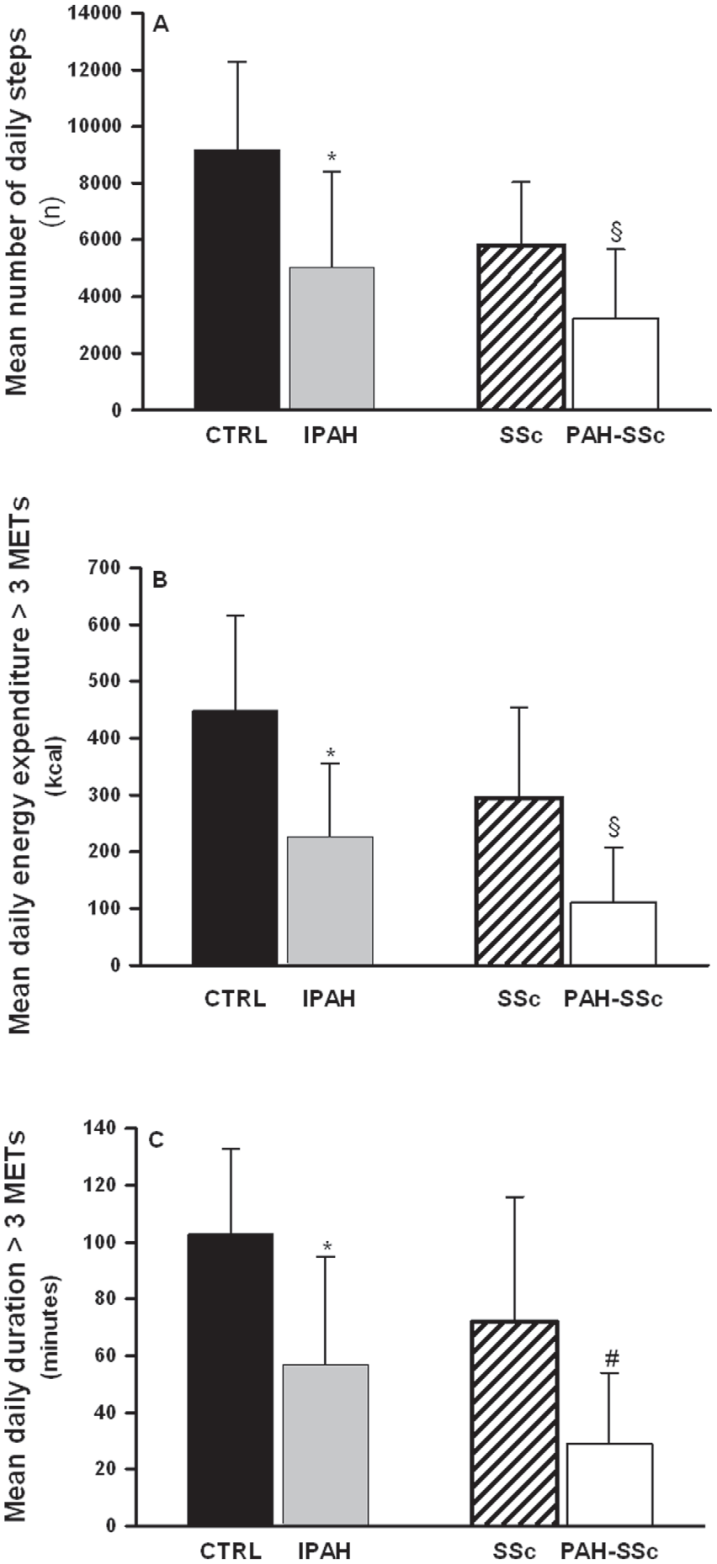
Daily life physical activities in pulmonary arterial hypertension patients and control groups. Mean number of daily steps (panel A), mean daily energy expended during physical activities inducing a metabolic demand >3 METs (panel B), and mean daily duration of physical activities inducing a metabolic demand >3 METs. (panel C) in healthy control subjects (CTRL; dark bars), patients with idiopathic pulmonary arterial hypertension (IPAH; grey bars), patients with limited systemic sclerosis without PAH (SSc; hatched bars), and patients with pulmonary arterial hypertension associated with limited systemic sclerosis (PAH-SSc; open bars). Values are mean ± SD. * p<0.01 vs. CTRL; § p≤0.01 vs. SSc; # p = 0.02 vs SSc. Figure legend: kcal, kilocalories; n, number.

The four groups demonstrated an equally good compliance with the physical activity monitor, wearing it for 12.9 (1.0), 12.5 (1.2), 11.9 (1.2), and 12.0 (2.0) hours/day for CTRL, IPAH, SSc and PAH-SSc respectively. As depicted in Figure 1A, IPAH and PAH-SSc patients presented significantly lower mean number of daily steps when compared with their respective controls (5041 (3357) vs. 9189 (3093) steps/day, p<0.01 for IPAH and CTRL respectively, and 3234 (2437) vs. 5810 (2226) steps/day, p = 0.01 for PAH-SSc and SSc respectively). PAH patients also showed significant decreases in their participation in physical activities inducing a metabolic demand >3 METs (energy expenditure and duration). (Figure 1B, 1C).

**Figure 2:**
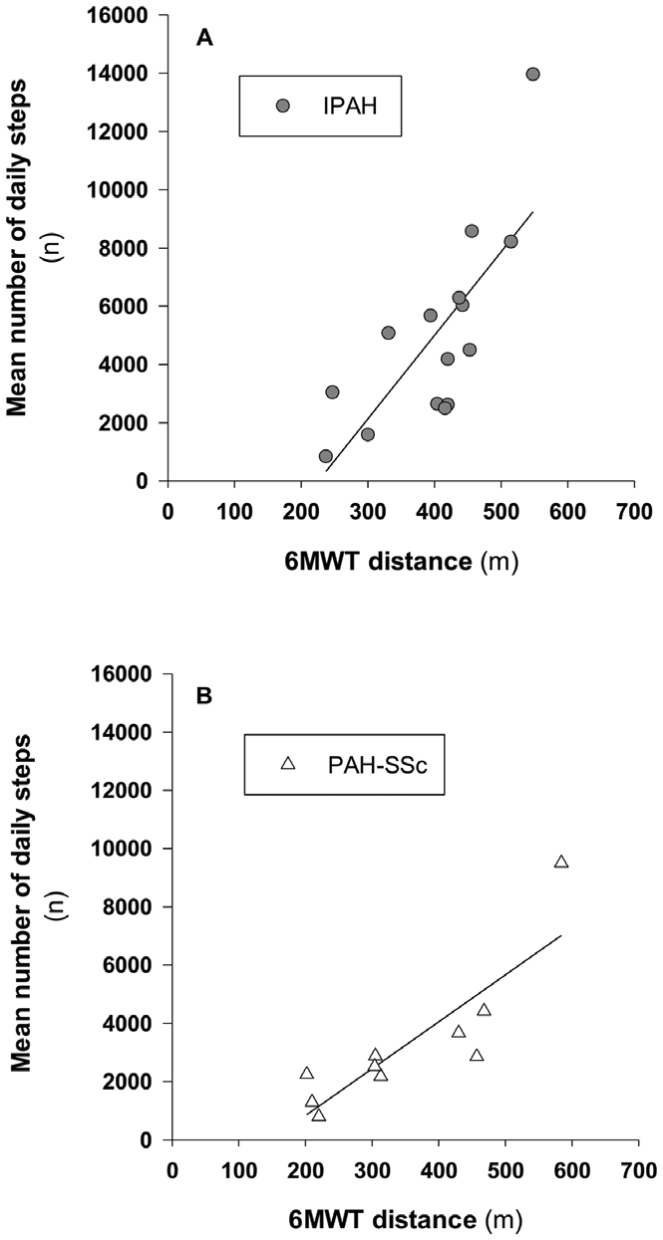
Correlation between exercise capacity and daily steps in pulmonary arterial hypertension patients. Correlation between the six-minute walk test (6MWT) distance and the mean daily steps in patients with idiopathic pulmonary arterial hypertension (IPAH; panel A) and in patients with pulmonary arterial hypertension associated with limited systemic sclerosis (PAH-SSc; panel B). Figure legend: n, number; min, minute.

**Figure 3:**
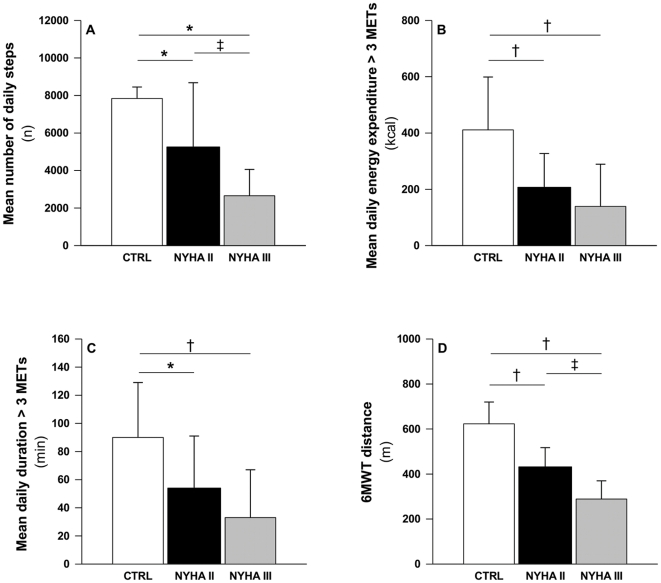
Relation between daily life physical activities and exercise capacity with the New York Heart Association functional class. Mean number of daily steps (panel A), mean daily energy expended during physical activities inducing a metabolic demand >3 METs (panel B), mean daily duration of physical activities inducing a metabolic demand >3 METs (panel C), and six-minute walk test (6MWT) distance (panel D) in healthy control subjects (CTRL; open bars) and in patients with pulmonary arterial hypertension in New York Heart Association (NYHA) functional class II (dark bars) and III (grey bars). Values are mean (SD). * p<0.05 and † p<.001 vs. CTRL; ‡ p<0.001 vs NYHA II. Figure legend: n, number; kcal, kilocalories; min, minute; m; meter.

The mean number of daily steps correlated with the distance walked during the 6MWT for IPAH and PAH-SSc patients (r = 0.76, p<0.01 and r = 0.85, p<0.01), respectively (Figure 2). Also, the energy expenditure and the duration of physical activities inducing a metabolic demand >3 METs correlated with the distance walked during the 6MWT amongst PAH patients (r = 0.52 and r = 0.52 for energy expenditure and duration respectively, both p<0.01). DL_PA_ were also progressively decreased with the worsening of NYHA functional class (Figure 3). Neither right atrial pressure nor cardiac index correlated with the 6MWT or with DL_PA_ in PAH (all p>0.30).

## Discussion

Compared to healthy controls and SSc patients, IPAH and PAH-SSc patients presented lower mean number of daily steps and lower participation to moderately intense physical activities (>3 METs). Amongst IPAH and PAH-SSc patients, DL_PA_ characteristics correlated with functional exercise capacity expressed by the distance walked during the 6MWT, suggesting that this test may be a surrogate of PAH patients' DL_PA_. The mean number of daily steps and functional exercise capacity decreased progressively as the NYHA functional class worsened. To our knowledge, this is the first study characterizing DL_PA_ and its relation with objective exercise capacity and NYHA functional class in PAH.

Exercise tolerance is generally assessed by the 6MWT in PAH. The distance walked during this simple and low cost test was found to correlate with patients' maximal oxygen consumption, NYHA functional class and survival [3]. The 6MWT is also thought to be representative of DL_PA_ in PAH, an assumption that has never been verified. This study confirmed that the 6MWT correlates with DL_PA_ in PAH. Among DL_PA_ characteristics, the mean daily steps strongly correlated with the distance walked during the 6MWT. Similarly, DL_PA_ decreased as NYHA functional class worsened. As the ultimate goal of PAH therapies is to improve patients' functional capacity in their daily life rather than the distance walked during the 6MWT *per se*, these results suggest that improvements observed during this test with PAH therapies could be clinically relevant for PAH patients. As the level of DL_PA_ is a parameter of increasing clinical interest and constitutes an important patient-centered outcome, whether changes in 6MWT observed in response to treatment translate into changes in DL_PA_ remains to be confirmed.

Despite recent improvements in long-term prognosis of PAH patients [1], a majority of patients displays persistent dyspnea and significant exercise intolerance. Exercise intolerance has been traditionally attributed to limited cardiac reserve, altered chronotropic response, ventilatory inefficiency and to ventilation/perfusion mismatch [23]–[24]. More recently, muscle dysfunction was pointed out as potentially contributing to exercise intolerance in PAH patients [25]–[27]. Although mechanisms leading to muscle dysfunction remain to be elucidated, inactivity may also contribute to exercise intolerance and muscle weakness in PAH. The present study documented lower DL_PA_ in PAH compared to matched controls even if the majority (64%) of the patients were in NYHA functional class II. According to the step-defined hierarchy documented by Tudor-Locke et al. [28], daily steps below 5000 and between 5000 and 7500 are representative of *«sedentary lifestyle index»* and *«low active»,* respectively. Thus, most PAH patients have adopted a sedentary lifestyle to a level comparable to older patients with severe chronic obstructive pulmonary disease (GOLD III) [18].

Because physical activity has been consistently associated with enhanced health related quality of life, interest to improve DL_PA_ is growing in several cardiorespiratory chronic diseases. Recent studies suggested that pulmonary rehabilitation in PAH may result in increased exercise capacity, and quality of life [29]–[31], and recent guidelines suggested that PAH patients may benefit from low graded aerobic exercise [32]. DL_PA_ may thus influence exercise capacity of PAH patients and explain the correlation between the 6MWT and the DL_PA_. It is noteworthy, however, that the effect of pulmonary rehabilitation on patients' DL_PA_ as well as the determination of which specific interventions and patients' characteristics are associated with more benefits and less risks remain unknown in PAH. Moreover, DL_PA_ may also be influenced by several non-physiological factors including emotional and psychological state, depression, anxiety, social support, body image and self-efficacy [33]-[34]. Those were not assessed in our study. Not surprisingly, DL_PA_ were not related to the distance walked during the 6MWT in controls, suggesting that DL_PA_ are more related to external factors than exercise limitation in controls. It also reinforces the concept that the 6MWT suffers from a ceiling effect that cannot discriminate the subjects with higher exercise and occupational capacities [8].

In conclusion, the participation in daily physical activities was reduced in PAH to a level that may contribute to further decrease exercise capacity. Moreover, the NYHA functional class and the 6MWT distance, which are routinely used as a markers of disease severity, prognosis and response to therapy in PAH, are also representative of patients' daily physical activity participation. Whether improvements in exercise capacity observed with PAH-specific therapies also increase the level of DL_PA_, an important patient-centered outcome, remain to be documented.
